# Force writes memory: proline isomerization as a molecular memory switch

**DOI:** 10.1042/BST20253127

**Published:** 2025-12-23

**Authors:** Ionel Popa, Ronen Berkovich

**Affiliations:** 1 Department of Physics and Astronomy, University of Wisconsin-Milwaukee, Milwaukee, WI, 53211, U.S.A.; 2 Department of Chemical Engineering, Ben-Gurion University of the Negev, Beer-Sheva, 8410501, Israel

**Keywords:** biophysics, intracellular signaling, learning and memory, mechanotransduction, protein dynamics, proline isomerization

## Abstract

Mechanical forces play a pivotal role in cellular processes, acting as molecular switches that encode, store, and retrieve information, thereby facilitating a form of molecular memory. This review explores how protein unfolding and refolding under tensile loads generate history-dependent responses that regulate domain stability and function. We focus on proline isomerization as a reversible switch, enabling distinct quasi-stable states that underpin medium- to long-term mechanical memory. Leveraging insights from molecular dynamics simulations and experimental data, we propose that proline isomerization creates a graded, adaptive memory response, distinct from binary on–off switches, with implications for biomaterial design and biorobotics. This mechanism offers a framework for developing force-responsive materials with memory properties, enhancing applications in tissue engineering and soft robotics.

## Introduction

Memory is a multistage process involving encoding, storage, and retrieval of information. Molecular changes in proteins and genes can establish stable, medium- to long-term memories if distinct stable states can be generated and the system can switch between these states. Mechanical forces can serve as such switches. Many proteins operating under mechanical load consist of numerous domains (often > 100, with bacterial counterparts exceeding 1,000), featuring slight sequence variations and stabilities. These proteins have a beads-on-a-string topology while tethered to other load-bearing structures (collectively termed polyproteins) [1,2]. As tensile forces build, some domains unfold, releasing additional contour length, while subsequent force reduction prompts refolding and shortening [3,4]. These dynamic changes in length maintain the experienced forces-per-molecule in a narrow range, typically below 20 pN [5,6]. This behavior resembles a computational bit-flipping mechanism, but without memory of prior cycles, unless an alteration modulates future responses.

During polyprotein unfolding under external force, the newly formed unstructured segments serve as flexible polymeric linkers that regulate the propagation of the mechanical tension to the remaining folded domains. The application of force introduces correlations between successive unfolding events [7], giving rise to a form of molecular ‘memory’ [8]. This memory is expressed through history dependence [9–11]: With each unfolding step, the linker lengthens, reducing the likelihood of subsequent unfolding and establishing a hierarchy of unfolding probabilities. As a result, progressively higher forces are required to unfold additional domains along the polyprotein chain [8,10–12].

Such mechanisms produce mechanically driven molecular memory, resulting not only from the fact that unfolding/refolding events add/subtract contour length during function but also from significant perturbations to the folding energy landscape. For example, it was proposed for muscle protein titin that unfolding can expose liable amino acids – such as cysteines – that can be posttranslationally modified, or covalent disulfide bonds that can be reduced. A posttranslational modification will typically destabilize domains, as addition of molecules crowds the folded core, and often refolding is impaired [13]. Disulfide reduction produces an increase in available contour length for future unfolding events, by freeing trapped sequence segments and completely changing the energy landscape of the protein [14]. However, both these processes are slow and frequently irreversible.

Additionally, protein–protein interactions offer another pathway for mechanical memory. Ligand binding often boosts mechanical stability, while dissociation resets it [15,16]. One such example is given by talin, a protein that regulates cell–extracellular matrix interaction [17,18]. Binding of ligands such as Deleted in Liver Cancer-1 (DLC1) or Rap1-GTP-interacting adaptor molecule protein (RIAM) to talin rod domains produces an increase in their mechanical stability. Hence, binding can tune the stability [19,20]. Talin also uniquely exposes through mechanical unfolding binding sites, recruiting proteins (vinculin) that lock the same rod domains open [21]. As these processes are easily reversible, but last significantly long time, they can be seen as a long-term molecular memory.

## The Proline Switch Mechanism

**Figure 1:**
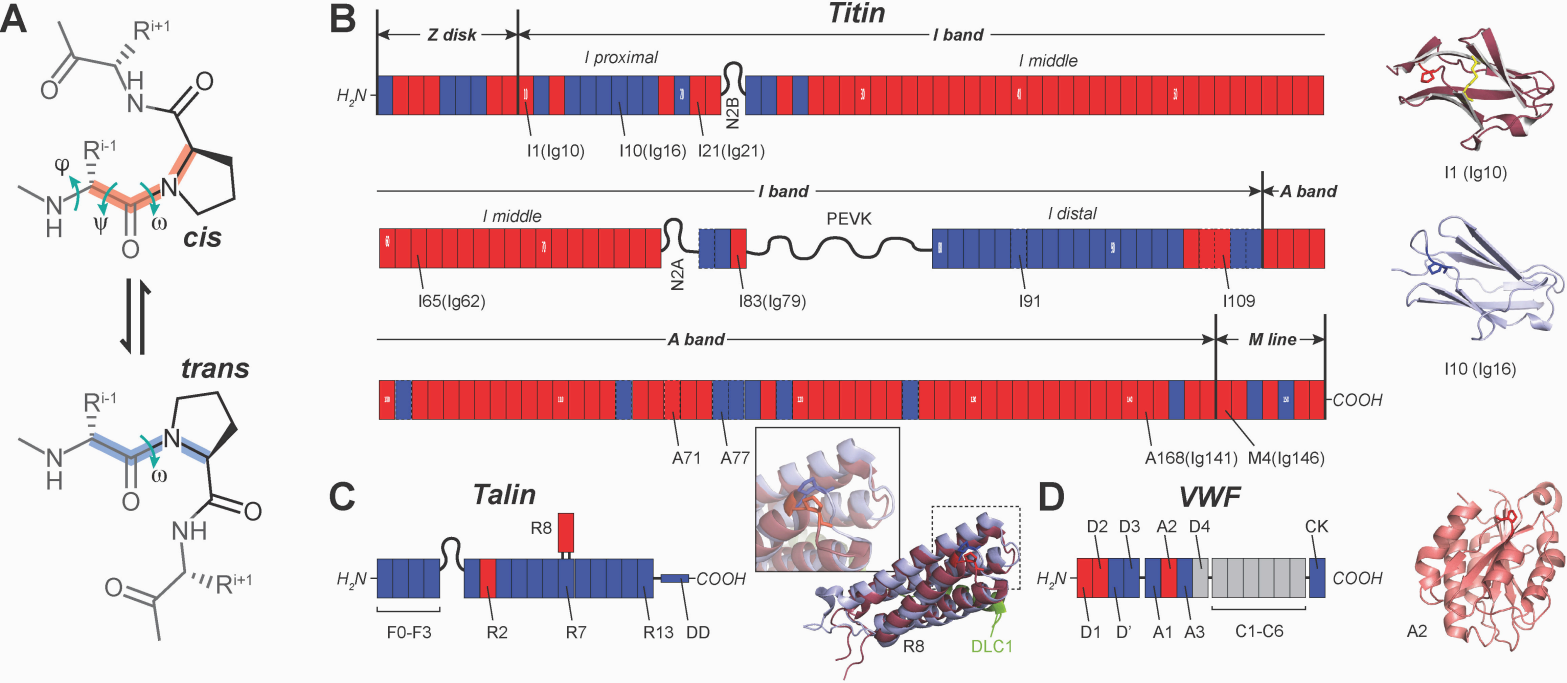
Proline switch as a short-term mechanical memory component. **(A**) Schematic of the cis-to-trans isomerization of proline, showing the torsion angles φ (N-C^α^ bond), ψ (C^α^-C bond), and ω (C-N peptidyl partial double bond). (**B**) (Left) Titin domain map (with each domain shown as a segment, and unstructured regions shown as a wavy line); red domains contain at least one cis-proline, while blue domains do not contain any cis-proline; the dotted domains are not predicted by UniProt ID: Q8WZ42-1 but have published structures in Protein Data Bank (PDB) database. (Right) Ribbon representation of domains I1 and I10, having a proline in cis and trans, respectively, shown as sticks (PDB: 1g1c and 5jdj). (**C**) (Left) Talin domain map; (right) structure of domain R8 non-bound and bound to DLC1 showing a proline switch (PDB: 2x0c and 5fzt). (**D**) (Left): von Willebrand factor (VWF) domain map; domains without known structure are shown in gray; (right) ribbon structure of domain A2 (PDB: 3gxb).

Another important mechanism, which will also be the focus of the remainder of this review, is the proline switch (Figure 1A). Because of the relatively small energy difference between the cis and trans conformations of the peptide bonds preceding proline residues (~1–3 k_B_T [22–24]), ~5% of prolines are in the cis form inside known folded structures [25]. While other amino acids can also have cis peptide bonds, their occurrence is thought to be insignificant (<0.05% [26]). The surprisingly low occurrence of cis-prolines, given the small energy difference between the isomers, implies that additional structural penalties or evolutionary selection further disfavor the cis state in most proteins. The spontaneous cis-to-trans isomerization of a proline peptide bond is a slow process under physiological conditions and is highly dependent on the adjacent amino acid sequence and environmental conditions. It was found to range from tens of seconds for the unfolded states to minutes (or even over 1 h) between folded states [27–33], allowing this process to act as a medium-term mechanical memory switch.

Under force, the proline switch gets activated as prolines that have the cis conformation are likely to transition to the trans form under force, given that this conformation is slightly more elongated [34,35]. As force decreases, the reverse cis-to-trans isomerization can occur before or after the acquisition of the tertiary structure. If folding occurred first, the new folded structure is expected to be less stable [36,37], while intrinsic dynamics will eventually produce the more stable cis conformation. However, if the protein experiences a second pull force before the trans-to-cis isomerization occurs, it will unfold at a lower force, due to this conformation-induced instability. Accordingly, we propose here that this proline switch can become a medium-term molecular memory variant, and its duration is related to the dynamics of the trans-to-cis isomerization while in the folded state. Furthermore, peptidyl-prolyl isomerase chaperones can act on the unfolded state, providing an additional tuning ‘knob’ [38,39].

## Proline Isomerization in Mechanosensitive Proteins

To gain further depth into how this mechanically triggered proline isomerization switch can provide a path for mechanical memory, next, we will consider three polyproteins operating under force: titin, talin, and von Willebrand factor (VWF).

Titin, the largest protein in the human body, is made of over 150 structured immunoglobulin (Ig)-like domains, predominantly composed of β-strands [40,41]. Titin connects the half-sarcomere bands of muscles and acts as both a molecular spring and an energy storage release. These functions are performed through unfolding and refolding of some of its IgG-like domains from the I-band – the elastic band of titin where there is no overlap between actin filaments and myosin motors. The I-band has entropic-spring regions (PEVK and N2B/N2A) and many IgG-like domains, varying based on muscle type. If we do a conformation analysis using known structures (determined experimentally or with AlphaFold [37]), we can identify which have at least one cis-proline in their native structure (Figure 1B). Surprisingly, over 70% of titin domains have at least one cis-proline. This is dramatically higher than the global ~5% cis frequency observed across all known folded structures [25]. The enrichment in titin suggests evolutionary selection for cis-prolines as regulatory mechanical switches rather than mere structural elements, consistent with their clustering in the mechanically active I-band. It seems that the majority of these domains are localized in the I-middle region, between N2B and PEVK. We note here that this region is typically the most alternately spliced [41]. Hence, it is likely that titin will also have different memory capabilities based on muscle type. Interestingly, the I-proximal region, which is thought to be the one containing the IgG domains that unfold and refold actively during contraction [40,42,43], has the fewest number of cis-proline. One exception is domain I1, which has a cis-proline and was recently shown to undergo force-induced isomerization [37]. This isomerization was associated with a switch between a strong and a weak state [37]. Interestingly, I1 domain can also form disulfide bonds, which adds an additional layer of complexity without changing the behavior of the proline switch [37,44]. While the domains from the middle region of titin unfold less often [40], we speculate that these unfolding events can switch them to the trans conformation. This switch would then enable the I-middle domains to unfold more frequently during the next relaxation-contraction cycles, if these cycles happen frequently enough. This assumption also agrees with the fact that the heart has the shortest titin isoform (and shortest I-middle region), as it needs to function in a very narrow set of parameters, while larger titin isoforms, such as the ones from leg muscles, need to quickly adapt to changing motion and switch their response based on the performed activity. This quick adaptation could be easily accomplished through the proline switch.

Talin is a multidomain protein that connects the cytoskeleton to the extracellular matrix and operates under force *in vivo* by unfolding and refolding its rod (R) domains. It has 13 R-domains, all α-helices, that can unfold and recruit up to 11 vinculin molecules to form novel connections to actin [17,18,45]. Back in 2022, using single-molecule magnetic tweezers, we reported a strange behavior that we measured for the R8 domain of talin, where under the same force, this domain was showing no unfolding transitions, overlapping folding-unfolding events with different kinetics, or no refolding transitions as a function of time [19]. Furthermore, what we noticed was that binding of a ligand (DLC1) to this domain locked talin R8 in a folded state, which can have major implications on regulating the mechanical activation of cells [19]. A quick structure analysis shows that this R8 domain [46], as well as domain R2 [47], has a cis-proline in their native state (Figure 1C). Interestingly, when analyzing structures where R8 was bound to DLC1 [48], or RIAM [49], the same proline is in a trans conformation. Hence, we reason that the cis-to-trans isomerization can fine-tune the mechanical response of some talin R-domains and provide a mechanism for mechanical memory. Furthermore, isomerization seems to be needed for successful binding of ligands, and this process is triggered *in vivo* by force. This suggests that a never-unfolded talin R8 domain will have a harder time recruiting a ligand compared with one that recently underwent an unfolding-refolding cycle. This mechanism might prevent binding of ligand to talin R8 before talin becomes part of the focal adhesion assembly. More recently, the talin R3 domain was also shown to have a force-induced mechanical hysteresis and a ~1 pN shift in unfolding force between successive cycles [50].

VWF is the largest blood protein, composed of repeated A, D, and C domains, and responds to transient shear forces developing in circulation to stop bleeding [51–53]. At least three of its domains have cis-prolines [54,55] (Figure 1D). VWF facilitates tension-activated platelet adhesion and aggregation at sites of vascular injury, while also stabilizing coagulation factors to support blood clot formation [56,57]. Domain A1 can bind receptors on platelet surface, while domain A3 can link to collagen, to initiate thrombosis [58]. Sandwiched in between, domain A2 contains a cryptic cleavage site for a disintegrin and metalloprotease with thrombospondin motifs 13 (ADAMTS13) protease, which is protected by its folded structure [59]. Under fluid shear stress, tension can build, leading to the unfolding and extension of domain A2, which can then be cleaved by ADAMTS13. Interestingly, domain A2 also contains a cis-proline [54], which may transition to a trans conformation under force. This transition can then either slow down refolding of A2, allowing more time for ADAMTS13-induced cleavage [54,60], or could produce a less stable folded structure, which would unfold at lower forces. Such a weak-folded structure would effectively represent a form of short-term mechanical memory, allowing VWF to remain active to ensure complete wound site recovery.

## Energy Landscape Perspective

Next, we will explore how the proline switch might function, from an energy landscape perspective. Protein folding is best understood through the lens of the energy landscape theory, which describes the protein’s potential energy surface as a rugged, funnel-shaped landscape [61]. The broad, high-energy top of the funnel represents the vast ensemble of unfolded conformations, while the narrow, low-energy bottom corresponds to the unique, thermodynamically stable native state. Folding is not a single, deterministic pathway but rather an ensemble of possible routes down this funnel, guided by a bias toward the native structure. The ‘ruggedness’ of the landscape is represented by transient traps or local energy minima where the protein can become kinetically stalled, and escape from these traps often constitutes the rate-limiting steps in the folding process.

**Figure 2:**
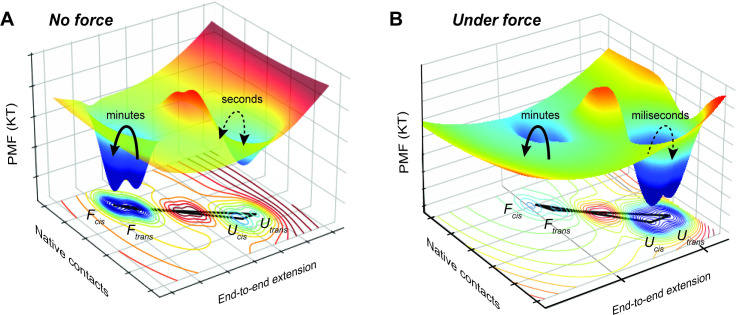
Schematic 3D potential of mean force(PMF) to illustrate force-dependent stabilization of proline isomers. **(A**) In the absence of force, the folded cis state, *F_cis_*, forms the deepest minimum, with *F_trans_* slightly shallower, while unfolded states *U_cis_* and *U_trans_* lie higher in energy. (**B**) Under force, folded states are destabilized, while unfolded states are stabilized, with *U_trans_* becoming the deepest minimum. The connectivity between minima reflects the possible transitions and demonstrates how applied force induces correlations between unfolding events, giving rise to mechanically driven molecular memory. Thick arrows indicate slow spontaneous isomerization (minutes to hours); thin dashed arrows indicate fast force-induced switching (milliseconds to seconds). The two-folded basins are therefore quasi-stable over minute timescales. In the absence of force, the cis-trans states of prolines in an unfolded peptide are only ~1–3 k_B_T apart, occurring with nearly equal probability.

In proteins containing proline, this picture becomes more complex (Figure 2). The unique structural duality of proline, its ability to exist in both cis and trans conformations, introduces four relevant minima with transition paths between them: *F_cis_* (folded cis state), *F_trans_* (folded trans state), *U_cis_* (unfolded cis state), and *U_trans_* (unfolded trans state). The folding process can thus proceed along four distinct paths: *F_cis_* ↔ *F_trans_*, *U_cis_* ↔ *U_trans_*, *F_cis_* ↔ *U_cis_*, and *F_trans_* ↔ *U_trans_*. Each of these has its own characteristic rate and barrier. Notably, the separation between *U_cis_* and *U_trans_* is largely force-independent, analogous to a chair-to-boat transition. Under equilibrium conditions without applied force, *F_cis_* is the deepest minimum, more stable than *F_trans_*, while *U_cis_* and *U_trans_* lie higher in energy, with *U*_*trans*_ slightly higher than *U_cis_*. When force is applied, however, the situation inverts: *F_cis_* and *F_trans_* are destabilized relative to the unfolded ensemble, while *U_cis_* and *U_trans_* become deeper minima, with *U_trans_* dropping below *U_cis_* and becoming the most stable state.

This behavior reflects a key physical principle: the trans conformation of a proline peptide bond is slightly more elongated than the cis conformation [34,35]. While this geometric difference may appear subtle, it has profound implications when the protein is subjected to mechanical stress. As force is applied, it preferentially stabilizes more extended states, effectively ‘tilting’ the energy landscape to favor stretched conformations. This mechanical bias actively drives the equilibrium toward the trans state, altering the protein’s folding routes and stabilizing conformations that would otherwise be less favored. The coexistence of cis and trans isomers in the unfolded state [62], therefore, fundamentally reshapes the folding landscape, producing a multiplicity of possible folding pathways.

The proline switch, thus, emerges as a sophisticated regulatory mechanism, balancing thermodynamic stability and kinetic control. Its function is rooted in the inherently low energy difference but slow interconversion kinetics between cis and trans, providing a basis for its role as a form of molecular memory [63]. While the underlying kinetics of the full state space remains Markovian, the inclusion of ultra-slow, force-triggered transitions – such as proline cis-trans isomerization – results in an effective breaking of ergodicity on the experimental timescales [8–10]. The system, therefore, appears non-Markovian when viewed within the conventional reduced state space (folded vs. unfolded). Hence, proline isomerization gives rise to mechanical memory: a prior unfolding event triggers a switch to a quasi-stable trans-folded state (*F*_*trans*_) with markedly different mechanical stability. Because the relaxation back to the native cis-folded state (*F*_*cis*_) is orders of magnitude slower than a typical folding/unfolding cycle, the system becomes kinetically trapped, preserving the history of the applied force [19,63]. This long-lived shift in the free-energy landscape functions as a reversible mechanical switch with memory. Furthermore, force exploits the subtle geometric difference between the cis and trans states to generate parallel unfolding pathways, effectively enabling a single polypeptide chain to exist in multiple mechanically stable native states [64]. The dramatic difference in unfolding forces between the cis and trans states of the titin Ig1 domain illustrates this principle, emphasizing how the proline switch underlies the function of mechanosensing proteins [37].

## Conclusion and outlook

The blueprint model linking cellular function of proteins to their sequence has deeply influenced the importance we give to the genetic code and DNA translation over post-expression changes and unfolding–refolding events. However, increasing evidence challenges this ‘code-to-fold-to-function’ linear view. Proteins with identical sequences were shown to adopt multiple conformations, exhibit distinct dynamics, and perform varied functions depending on their cellular context, environmental conditions, or post-translational modifications [13,14,65–72]. This conformational heterogeneity provides an added layer of adaptability, enabling rapid complex responses to changing environments. Such fast adjustments are particularly critical for force-bearing proteins, which are often too large to be fine-tuned through regular turnover and must instead rely on dynamic conformational shifts to cope with irregular mechanical demands.

Here, we looked at proline isomerization as a potential memory mechanism for three protein systems. Titin shows a surprisingly high occurrence of cis-prolines in its folded domains (>70% compared with ~5% for typical proteins), most of them localized in the middle of its elastic I-band. This region is thought to have intermediate stability [40], which is ideal for medium-term memory, while the proximal I-domains that unfold first during muscle contraction do not require this added level of complexity. Furthermore, as its name suggests, the unstructured PEVK region of titin is rich in proline amino acids, and how isomerization might influence its function remains an open question. Talin has two R-domains with cis-proline in their folded structure, and the isomerization of domain R8 seems to ensure that recruitment of ligands only takes place when tethered, following an unfolding-refolding event. The A2 domain of VWF features a cis-proline that may isomerize to trans under shear stress, potentially delaying refolding to prolong ADAMTS13 cleavage or creating a less stable state for enhanced responsiveness in hemostasis, thereby encoding a short-term memory of vascular injury events.

While here we discussed only these three systems, we think that many more molecular processes can be described in a similar way. Furthermore, for proteins operating under force, we can envision other mechanisms for mechanical memory. Kinetic trapping into meta-stable states [16,73], domain misfolding and interdomain misfolded structures [74–76], and domain swapping [77,78] can provide a mechanism for memory, albeit even shorter term or less reproducible. Another pathway might come from fold switchers, which are proteins that have one native fold in the cytosol and another at the plasma [79–82]. However, such a behavior has yet to be determined for force-bearing proteins.

Understanding mechanical memory can play a role not only in deciphering how the human body works but also in developing novel biomaterials with memory properties. For example, biomaterials can be produced to have globular proteins that maintain their folded structure and are surrounded by a similar water environment as in the cells [83]. These protein hydrogels display similar responses to mechanical forces as tissues, as they have intrinsic visco-elasticity directly related to unfolding/refolding events [84]. Here, the proline switch could enable tunable memory, allowing them to ‘remember’ mechanical stress through reversible cis-trans isomerization of peptide bonds, which alters domain stability and refolding kinetics without irreversible chemical changes. Given the complexity of the protein-network system [85], we predict a more graded, dial-like response rather than an on–off switch, producing adaptive responses, such as self-healing and actuated motion.

**Perspectives**

- Mechanical forces serve as dynamic switches in polyproteins, encoding and storing memory through unfolding and refolding events, maintaining tensile forces within a narrow range (<20 pN), and establishing a history-dependent hierarchy of domain stability that mimics computational bit-flipping mechanisms.
- The proline switch can play a key role in medium-term mechanical memory for protein domain with native cis-prolines, which can be turned into their trans-isomerization state during unfolding and generate weaker native states that are more prone to unfold again, at lower force.
- Understanding mechanically driven molecular memory, controlled by proline switches, will inspire the development of novel artificial tissues and biomaterials with adaptive memory properties. Future directions include leveraging this mechanism to design smart biomaterials that mimic biological resilience, such as self-healing scaffolds for tissue engineering or force-responsive implants that adapt to physiological loads. Integrating proline-based switches into biorobotics could enhance robotic motion control, enabling adaptive locomotion in dynamic environments, like soft robots navigating uneven terrain.

## Open Access

This article has been published open access under our Subscribe to Open programme, made possible through the support of our subscribing institutions, learn more here: https://portlandpress.com/pages/open_access_options_and_prices#conditional

## Abbreviations

ADAMTS13: a disintegrin and metalloprotease with thrombospondin motifs 13
DLC1: Deleted in Liver Cancer-1
PDB: Protein Data Bank
PMF: potential of mean force
RIAM: Rap1-GTP-interacting adaptor molecule protein
VWF: von Willebrand factor
